# Fear of Falling and Subjective Cognitive Decline in Racially Diverse Adults Aged 60+ in Low-Income Communities

**DOI:** 10.1093/geroni/igaf122.3771

**Published:** 2025-12-31

**Authors:** Renata Komalasari, Nikki Hill, Ayse Malatyali, Janet Lopez, Sergi Garcia-Retortillo, Jian Zou, Veronica Decker, Ladda Thiamwong

**Affiliations:** Akademi Keperawatan Andalusia Jakarta, Jakarta, Jakarta Raya, Indonesia; The Pennsylvania State University, University Park, Pennsylvania, United States; University of Central Florida, Orlando, Florida, United States; University of Central Florida, Orlando, Florida, United States; University of Central Florida, Orlando, Florida, United States; University of Central Florida, Orlando, Florida, United States; University of Central Florida, Orlando, Florida, United States; University of Central Florida, Orlando, Florida, United States

## Abstract

Subjective cognitive decline (SCD) and perceived falling or fear of falling (FOF) share similar mental health consequences. Yet, few studies have explored their relationship in racially diverse older adults. This study examined associations between SCD, SCD-related functional difficulties, and FOF, along with the moderating effects of depressive and anxiety symptoms in 91 community-dwelling adults aged 60 + (Mean age = 73.26, SD ± 6.99; 89% female; 96.7% non-White) in Central Florida. FOF was measured using the short Fall-Efficacy Scale International. SCD was assessed using a single question on confusion or memory loss, with a follow-up Likert-scale item for functional difficulties. Depression and anxiety were measured using validated screening tools, including the Patient Health Questionnaire-9 (PHQ-9) and Geriatric Anxiety Inventory-Short Form. We found that SCD, SCD-related functional difficulties, and anxiety symptoms were not significantly associated with FOF, but depressive symptoms were (e.021= 1.021, p=.020). A Poisson regression analysis examined associations between the variables and FOF. Higher depressive symptoms were associated with higher FOF. The interaction of SCD-related functional difficulties with depressive symptoms (e-.002= .998, p=.843) was not associated with FOF, but its interaction with anxiety symptoms was (e.073= 1.075, p=.035). The association between SCD-related functional difficulties and FOF was accentuated as anxiety level increased and attenuated with lower anxiety symptoms. Older adults with more depressive symptoms report greater FOF. Anxiety amplifies the link between FOF and functional difficulties due to subjective cognitive decline. Future research should investigate how FOF and emotional well-being evolve as cognitive deterioration progresses and daily functioning becomes increasingly impaired.

